# Plasma 11-deoxycorticosterone (DOC) and mineralocorticoid receptor testicular expression during rainbow trout *Oncorhynchus mykiss *spermiation: implication with 17alpha, 20beta-dihydroxyprogesterone on the milt fluidity?

**DOI:** 10.1186/1477-7827-6-19

**Published:** 2008-05-19

**Authors:** Sylvain Milla, Xavier Terrien, Armin Sturm, Fidaa Ibrahim, Franck Giton, Jean Fiet, Patrick Prunet, Florence Le Gac

**Affiliations:** 1 Institut National de la Recherche Agronomique, INRA-SCRIBE, IFR 140, Campus de Beaulieu, 35000 Rennes, France; 2 The University of Namur (FUNDP), Unité de Recherche en Biologie des Organismes (URBO), 61, rue de Bruxelles, 5000, Namur, Belgium; 3 The University of Stirling, Institute of Aquaculture, Stirling, FK9 4LA, Scotland, UK; 4 AP-HP, Laboratoire de Biologie Hormonale, hôpital Saint-louis, Paris, France; 5 AP-HP CIB SUD, INSERM IMRB U841eq07, hôpital Henri Mondor, Faculté de Médecine, 94010 Créteil, France; 6 INSERM IMRB U841eq07, Hôpital Henri Mondor, Faculté de Médecine, 94010 Créteil, France

## Abstract

**Background:**

In rainbow trout (*Oncorhynchus mykiss*), the endocrine control of spermiation is not fully understood. Besides 11ketotestosterone (11KT) and 17alpha, 20beta-dihydroxyprogesterone (MIS), the potential physiological ligand of the mineralocorticoid receptor (MR) 11-deoxycorticosterone (DOC), is a credible candidate in *O. mykiss *spermiation regulation as spermiation is accompanied with changes in aqueous and ionic flows.

**Methods:**

In this study, we investigated potential roles of DOC during spermiation 1) by describing changes in blood plasma DOC level, MR mRNA abundance during the reproductive cycle and MR localization in the reproductive tract 2) by investigating and comparing the effects of DOC (10 mg/kg) and MIS (5 mg/kg) supplementations on sperm parameters 3) by measuring the in vitro effect of DOC on testis MIS production.

**Results:**

The plasma concentration of DOC increased rapidly at the end of the reproductive cycle to reach levels that were 10–50 fold higher in mature males than in immature fish. MR mRNA relative abundance was lower in maturing testes when compared to immature testes, but increased rapidly during the spermiation period, immediately after the plasma rise in DOC. At this stage, immunohistochemistry localized MR protein to cells situated at the periphery of the seminiferous tubules and in the efferent ducts. Neither DOC nor MIS had significant effects on the mean sperm volume, although MIS treatment significantly increased the percentage of males producing milt. However, a significant reduction in the spermatocrit was observed when DOC and MIS were administrated together. Finally, we detected an inhibitory effect of DOC on testis MIS production in vitro.

**Conclusion:**

These results are in agreement with potential roles of DOC and MR during spermiation and support the hypothesis that DOC and MIS mechanisms of action are linked during this reproductive stage, maybe controlling milt fluidity. They also confirm that in *O. mykiss *MIS is involved in spermiation induction.

## Background

In rainbow trout *Oncorhynchus mykiss*, "spermiation" is the reproductive stage when hydrated sperm released from testis to the vas deferens can be collected manually from male fish. The fish endocrine control of spermiation is complex and not fully understood. A large number of studies suggest that secretion of GnRH (Gonadotropin Releasing Hormone) increases circulating gonadotrophins (GtHs, today considered as FSH and LH) which in turn triggers fish spermiation. Indeed, a role of GtHs in spermiation has been suggested by its reported ability to generate or increase sperm production, milt hydration and spermatozoa excretion [1-3]. According to the current model, GtHs either act directly on seminiferous tubules or sperm ducts or its effects are mediated via changes in gonadal steroids (see for review [4]). In this way, exogenous treatments with androgen steroids induced precocious spermiation in goldfish *Carassius auratus *[5]. In salmonids, androgens show the highest levels when sperm excretion begins [6,7] but fall while the production of sperm develops [6,8]. However, androgens were not effective to induce spermiation in amago salmon *Oncorhynchus rhodorus *[9]. These studies show that androgens are implicated in the hormonal regulation of salmonid spermatogenesis, however, their role in "spermiation" is unclear.

The steroid hormone 17alpha, 20beta-dihydroxyprogesterone has been dubbed MIS "Maturation-Inducing Steroid" because of its effects in females of several teleost species. As other sexual steroids, MIS production is stimulated by the administration of GnRH and GtHs *in vivo *and *in vitro *[1,9-11]. High plasma levels of MIS are measured in mature salmonids [9,12,13] and a significant correlation between the plasma level of MIS, the production of sperm and the ionic concentrations in the seminal fluid was reported in some salmonids and Atlantic halibut *Hippoglossus hippoglossus *[6,9,11,14]. However, studies where MIS has been administered to mature male fish led to a range of effects on spermiation. Injections of this steroid induced precocious spermiation in the salmonids brook trout *Salvelinus fontinalis *[15] and *O. rhodorus *[11] and stimulated milt production in Japanese eel *Anguilla japonica *[16], *Pagrus auratus *[10] and *Carassius auratus *[12]. In other non-salmonid species, similar treatments suggest a role of this steroid in the milt hydration [14,16,17]. However, injections of MIS into spermiating *O. mykiss *or non-spermiant Atlantic salmon *Salmo salar *did not modify the volume of sperm [18,19] but affected the ionic composition of the seminal fluid [19]. Taken together, most results suggest that MIS is involved in the control of sperm excretion, yet its particular role in the endocrine control of spermiation in salmonids is far from clear.

Throughout the spermiation in *O. mykiss*, the increase in milt production and hydration is accompanied with changes in seminal fluid ionic concentrations [6,8]. Although such changes in water and ions flux through the seminiferous tubule or sperm duct epithelia could also involve control by the mineralocorticoid endocrine system, this hypothesis has received little attention. In mammals, the mineralocorticoid aldosterone mediates its effects on epithelia involved in the control of the hydromineral balance via interaction with the mineralocorticoid receptor (MR) [20]. Intriguingly, aldosterone effects have further been reported on sodium and water absorption in mammal seminiferous tubules, testis and epididymis [21,22]. In contrast to mammals, reliable evidence of aldosterone in teleost fish is lacking, and recent molecular data indicate that teleost genomes do not have an orthologue of human CYP11B2, the enzyme catalysing the last step of aldosterone biosynthesis [23,24]. Interestingly, however, teleosts possess a mineralocorticoid receptor that has been initially isolated from a testis cDNA library [25] and prefers mineralocorticoids over glucocorticoids. 11-deoxycorticosterone (DOC) is a corticosteroid with high affinity to the *O. mykiss *MR [26] and was detected at substantial level in a milt producing male *O. mykiss *[27]. In view of the established roles of aldosterone in mammals, we hypothesize that the rtMR and its potential ligand DOC could play a role in teleost male reproduction, which could explain the high plasma level of DOC previously observed in a spermiating male *O. mykiss*.

Thus, in addition to MIS, DOC requires further research on its possible involvement in the endocrine control of spermiation in *O. mykiss*. To assess the effects of DOC during spermiation and its potential interactions with the effects elicited by MIS 1) we measured the seasonal variation of plasma DOC and rtMR mRNA levels 2) we localized rtMR in the testis and the vas deferens 3) we examined the effects of DOC and MIS, alone or together, on milt features. 4) Finally, to investigate whether DOC could act indirectly on sperm hydration by influencing testis steroidogenesis, we studied *in vitro *the effect of DOC on testis MIS production.

## Methods

### Tissue collection for detecting seasonal variation of plasma 11-deoxycorticosterone (DOC) and MIS, and rainbow trout Oncorhynchus mykiss mineralocorticoid receptor (rtMR) mRNA expression

Investigations and animal care were conducted according to the guidelines for the use and care of laboratory animals and in compliance with French and European regulations on animal welfare.

In a first experiment, male *O. mykiss *were reared at the INRA experimental fish farm PEIMA (Britanny, France) under natural temperature and photoperiod. Male *O. mykiss *from an autumn spawning strain were sampled at different time points between September and May for the "immature" fish, and from September to December for the "maturing" fish (in this group of fish, the mature males had been eliminated in December). Blood plasma was collected and stored at -20°C pending DOC measurements. Fish were stripped to detect spermiation, a piece of each gonad was fixed in Bouin's fixative for histological examination of spermatogenesis; the stage of testicular development was determined as previously reported [28]. Another piece was frozen in liquid nitrogen and kept at -80°C until RNA extraction for rtMR mRNA quantification.

In a second experiment, maturing male and immature *O. mykiss *from a spring spawning strain were sampled once a month from January to July, and blood plasma were used for DOC and MIS measurements. At each sampling time fish were stripped by abdominal pressure and sperm production was evaluated according to the following "index of spermiation": **0 **: no milt could be collected ; **1**: 10 to 100 μl, **2 **: 0.1 to 1 ml, **3 **: >1 ml of milt.

### Steroid supplementation *in vivo*

Two year old male *O. mykiss *from a spring spawning strain from PEIMA experimental fish farm were held in a recirculating water system under natural photoperiod at 12°C. On day 0 of the experiment, males were just before or at the early beginning of spermiation. Fish were anaesthetized in 0.03% 2-phenoxy-ethanol, weighted and implanted intraperitoneally with DOC (10 mg/kg) or MIS (MIS, 5 mg/kg) or the combination of these two hormones in silastic implants : ref 1707680, Silastic (R) MDX4-4210 Biomedical Grade Elastomer with catalyst (Dow Corning Corporation, Midland, USA). These doses are classically used for fish steroid implantation. Fish were sampled 2, 9, 16, 23 and 41 days after implantation. Blood samples were obtained from 4 implanted fish in each group for steroid measurement. Milt production was checked and when possible (more than 0.5 ml of sperm collected per stripping), milt volume was measured and aliquots were used to measure spermatocrit, pH and sperm motility. Seminal fluid was then separated from spermatozoa by centrifugation at 1000 g for 10 min at 4°C for measurements of seminal fluid osmolality and sodium/potassium concentration.

### Blood plasma 17,20β-P and DOC measurements

MIS levels were measured in plasma and culture media by radioimmunoassay as previously described [29]. DOC levels were measured in plasma with radioimmunoassay (RIA) using two extraction/chromatographic procedures. In experiment 1, in order to monitor losses occurring during the extraction, chromatography and redissolution steps before immunoassay, a tracer dose of 2000 dpm of tritiated DOC was added to the blood plasmas prior extraction. Extraction was carried out with 15 ml of ethyl acetate + 0.1% triethylamine (TEA). The organic phase first evaporated, was redissolved in 1 ml of a mixture of isooctane + dichloromethane 98/2 (v/v) + 0.1% of TEA, then vortexed 60 s, ultrasonicated 10 min and vortexed again. This organic mixture was layered onto the Celite column [30], then we successively added onto the column 5 ml of pure isooctane, then 5 ml of a mixture of isooctane/dichloromethane 96/4 (v/v) + 0.1% TEA without collecting [31]. Finally the DOC fraction was eluted with 5 ml of a more polar mixture of isooctane/dichloromethane 88/12 + 0.1% TEA. This last fraction was evaporated, and the dried residue was redissolved in phosphate gelatin buffer (1 ml). Then 100 μl of extract were incubated with 100 μl of [^3^H] DOC (American Radiolabelled Chemicals, 10000 cpm), 100 μl of rabbit anti-DOC antibody diluted 1/5000 previously validated [32]. The samples were vortex-mixed, then incubated at room temperature for 15 to 20 h under rotating agitation before being counted with a beta-ray counter. In experiment 2, a second RIA protocol with some modifications was used with the same antibody. The steroid extraction was carried out with cyclohexane/ethyl acetate (50/50, v/v) and HPLC was used to separate DOC from the other steroids (column ZORBAX C18 4.6*250 mm, ref. 880975-902, Agilent, Massy, France; mobile phase: acetonitrile/eau, 80/20, v/v). In addition of the main plasma steroids tested for their cross-reactivity with the antibody [32], cortisone (a cortisol precursor detected in fish plasma) and MIS cross, were also tested. For both steroids, the cross reactivity was lower than 0.01%. No other steroids had a cross reactivity higher than 1.66 % (corticosterone). In both assays, DOC RIA was carried out in duplicate.

### Total RNA extraction, reverse transcription and Real-time PCR

Total RNA was extracted from the testis and vas deferens tissues using the TRIzol reagent (Invitrogen, Cergy-Pontoise, France). The RT PCR protocol has been described elsewhere [33]. Reverse transcription was performed using 2 μg of total RNA according to the following procedure (Promega, Madison, WI, USA). After RNA denaturation and a 10-min step at 30°C, reverse transcription was performed at 37°C for 60 min. Real-time PCR analysis was performed using a SYBR Green PCR Master Mix (Eurogentec, Seraing, Belgium) using 600 nmol l^-1 ^of primers. Amplification parameters were as follows: each of the 40 cycles consisted in 15 s of denaturation at 95°C, and 40 s annealing/extension at 60°C. Ribosomal 18S mRNA abundance was determined for use as an internal standard. Primer sequences, Genbank accession number of the target gene and PCR product sizes are presented in table 1. A melting curve analysis was performed to verify that a single PCR product was generated. Negative controls, performed by omitting reverse transcriptase from the RT step, remained consistently negative.

**Table 1:** Nucleotide sequence of RT-PCR primers, Genbank accession number, and PCR product size for target genes

**Gene**	**Genbank**	**Forward sequence**	**Reverse sequence**	**Size**
rtMR	AF209873	GAAACAGATGATCCGCGTGGT	TGGATCAGGGTGATTTGGTCCT	87
18S	AF309412	CGGAGGTTCGAAGACGATCA	TCGCTAGTTGGCATCGTTTAT	92

### MR Immunohistochemistry

To generate an antigen for antibody production, a part of the N-terminal domain of the rtMR was expressed as a fusion protein with *Schistosoma japonicum *Glutathione S-transferase (GST), using a commercial system (Amersham). Using appropriate primers and enzymes, a cDNA was generated by PCR and subcloned in frame into the vector pGEX-6P-1 to generate a fusion protein in which amino acids 12 to 228 of the rtMR are added to the C-terminus of GST. The fusion protein was expressed in a suitable bacterial strain (*E. coli *BL21-codon Plus (DE3)-RP, Stratagene) and purified from cell lysates by affinity chromatography on GSH-sepharose. The isolation of a protein of a GST-fusion protein of the expected molecular weight was confirmed by Western Blot using an antibody against GST (Amersham). The antigen was emulsified with complete Freund's adjuvant and used to immunize rabbits. Serum was collected after 5 antigen injections and before immunization.

For rtMR localisation, pieces of testes in stage VII-VIII of development (the beginning of spermiation) of development and vas deferens (stage VII) were sampled for rtMR immunolocalization. Tissues were fixed in 4% paraformaldehyde with acetic acid for 16 h at 4°C and then embedded in paraffin. Sections were cut and mounted on polylysine slides. Antigen retrieval was carried out by microwave treatment for 15 min in 10 mM (pH 6.0) citrate buffer. The sections were immunocytochemically stained by the biotin/streptavidin/peroxidase complex (HRP/DAB) method using a commercial kit (Labvision corporation, Fremont, CA). The primary antiserum diluted 1:1250 was applied to sections at room temperature for one hour. The specificity of the immunoreaction was confirmed by incubating other sections with preimmune serum at the same dilution or without the second antibody.

### Western blot for antibody specificity

Specificity of the antiserum raised against rtMR was checked by transiently expressing rtMR in COS-7 cells and carrying out Western blot on extracts from transfected cells. Immunoreactivity was observed with a protein fraction of the expected molecular mass in samples from rtMR-expressing COS-7, but not in untransfected COS-7 cells, showing the specificity of the antibody.

### Spermatocrit, sperm pH and spermatozoa motility; fluid seminal osmolality and sodium/potassium concentration

Milt samples were centrifuged in hematocrite tubes at 12620 g for 15 min (centrifuge 201 m, Sigma-Aldrich, Saint-Quentin, France) and the spermatocrit was measured from the ratio of spermatozoa pellet to total milt volume. Sperm pH was measured with an electronic pH meter with a spear electrode. Sperm motility was determined by subjective estimates of percentage of motile sperm cells and by the duration of mobility. Milt samples were diluted 1/200 in a seminal fluid mimicking medium (SFMM see [34]) prior activation by dilution 1/20 in insemination fluid directly under the microscope. Sperm motility was assessed by evaluating the percentage of motile spermatozoa immediately (~3 sec.) and 20 seconds after activation. Seminal fluid osmolality was measured with an osmometer (Bioblock, Vanves, France). Sodium and potassium concentrations were measured using a flame photometer (VWR international, Fontenay-sous-bois, France) after diluting the samples to 1/400 for sodium and to 1/200 for potassium.

### *In vitro *testis incubation and hormonal treatments

Gonads of 4 individual males in the pre-spermiation stage were pooled and cut in ~2 mm^3 ^fragments; testicular fragments were washed in L-15 incubation medium, and subsequently incubated for 24 hours at 12°C under gentle agitation (50 RPM) in 24-well culture plates. In each well were added 5 fragments of testis per 0.5 ml of L15 culture medium (pH 7.7) supplemented with hepes, 4,76 g/L; Na_2_(CO_3_), 413 mg/L; CuCl, 500 μg/L; MnSO_4_, 0.1 mg/L; Se(NaSeO_3_), 50 mg/L; lactic acid, 110 mg/L; L-glutathione reduced 1.610^-5^M and E vitamin 0.3 M. DOC or cortisol (1, 10 and 100 ng/ml), were added in 1 μl ethanol vehicle, alone or in combination with GtH (30 ng/ml). Controls received vehicle alone and each treatment was tested in triplicates. After incubation, the culture media were collected for MIS measurement.

### Statistical analysis

The seasonal variations in blood plasma DOC and testis rtMR RNA abundance, changes in blood plasma steroid concentrations after hormone implants, as well as the effects of hormonal treatments on MIS *in vitro *production, were analyzed by non-parametric Kruskall-Wallis tests as data violated assumptions of normality and/or homogeneity of variance. The hormonal treatment effects on spermiation induction (percentage of fish producing milt) were analyzed using a Chi^2 ^test. The effect of sampling time and of treatment on other milt parameters were analyzed using a two-way analysis of variance ANOVA (carried out on arcsine square root transformated data) followed by a post-hoc Scheffe test to determine differences between dates or between hormonal treatments.

## Results

### Seasonal variations of plasma 11-deoxycorticosterone (DOC)

**Figure 1:**
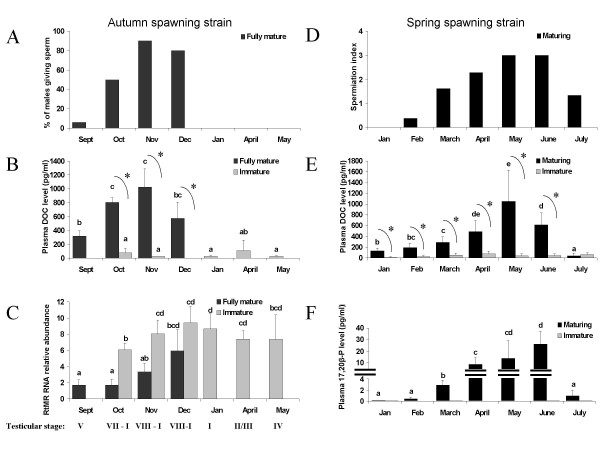
**Plasma DOC and MIS variations during the *O. mykiss *reproductive cycle. A, B, C : experiment 1**. with *O. mykiss *from an autumn spawning strain. Kinetics of initiation of spermiation in a group of mature males, expressed as % of fish producing milt (A). Coincident changes in blood plasma DOC concentrations (pg/ml) (B) and in testis mineralocorticoid receptor (rtMR) mRNA abundance (C) according to time of year and stage of sexual maturation. Means+ SD (n = 3–5). Mature males were only studied until December. **D, E, F : experiment 2 **with *O. mykiss *from a spring spawning strain. Kinetics of spermiation in a group of mature males, assessed by the spermiation index (D). Coincident changes in blood plasma DOC concentrations (pg/ml) (E) and in blood plasma MIS (ng/ml) (F) according to time of year. Means+ SD (n = 8). Different letters indicate significant differences between months. * Significant difference between immature and maturing/mature fish, p < 0.05.

In the first experiment using autumn spawning strain, spermiation started in September and the maximum number of males producing milt was reached in November (Figure 1A). Figure 1B shows the variation in blood plasma DOC concentrations during the reproductive cycle in this strain. In the immature males (stage I), the average DOC level was low in October and November and remained at low levels (20–100 pg/ml) until May. In the maturing fish, DOC level were elevated in September, peaked at about 1 ng/ml in November (stage VIII, full spermiation) – corresponding to levels 10–50 fold higher than in immature fish (p < 0.05) then tended to decline in December (about 600 pg/ml – not statistically significant). In this group of fish, the mature males had been eliminated in December.

Results from a second experiment using a spring spawning strain confirmed the occurrence of a significant increase in plasma DOC levels at the end of the reproductive cycle (Figure 1E). The DOC values peaked in May in fully mature males (mean = 1048 pg/ml; range = 240 to 1800 ng/ml) whereas DOC levels remained low in immature males over the whole experiment (mean = 43 +/- 23 pg/ml). The 8-fold rise between January and May coincided with an increase of the spermiation index (as defined in Methods, Figure 1D). A large increase of MIS plasma concentrations was also observed in mature males and this steroid was also very low or undetectable in immature males (Figure 1F). The amplitude of the MIS increase was larger (200-fold increase between January and June), while that of DOC appeared to start earlier during the annual cycle, with DOC levels being significantly higher in mature than in immature male fish already in January (p < 10^-3^).

### Rainbow trout *O. mykiss *mineralocorticoid receptor (rtMR) mRNA abundance and rtMR protein localization in the reproductive tract

Using RT-PCR the transcript for rtMR was detected in testicular tissues. At the beginning of the reproduction period (september-november in the autumn strain), rtMR mRNA relative abundance was significantly lower in mature fish than in immature fish (Figure 1C) (p < 0.05). The rtMR transcripts increased from October to December, and the rise was more pronounced in mature males (3-fold) than in immatures (1.5-fold). From November to May, rtMR mRNA levels remained relatively constant in immature gonads or in early stages of maturation.

Anti-rtMR antibody obtained as specified in Methods was tested by Western blot analysis using protein extracts from COS-7 cells transiently expressing rtMR. The anti-rtMR antibody recognized a 102 to 112 kDa band corresponding to rtMR expected molecular mass. The pre-immune antibody was used as a negative control and was unable to recognize this band.

**Figure 2:**
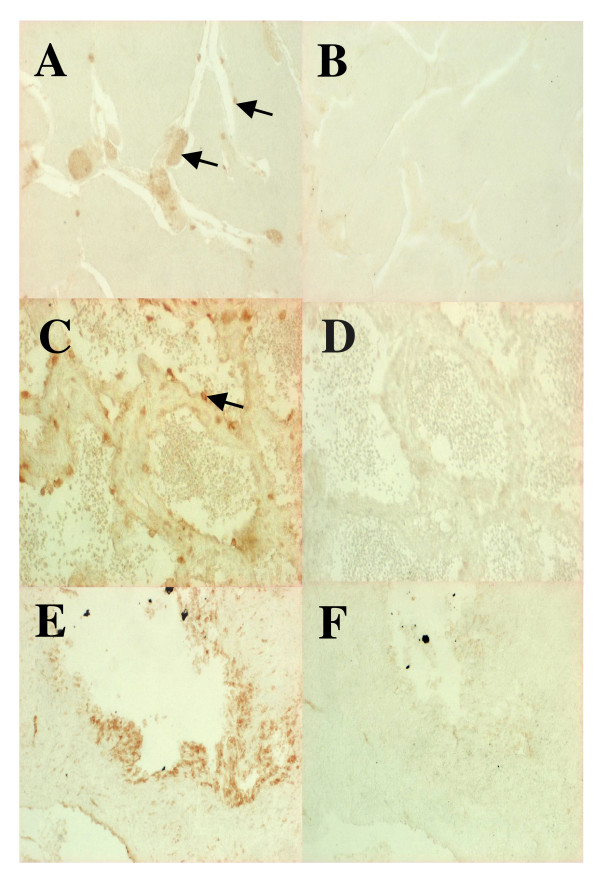
**Immunocytochemical localization of mineralocorticoid receptor (rtMR) in *O. mykiss *gonads**. Gonads were incubated with a primary antibody directed against the rtMR A/B domain (A: testis stage VII; C: testis stage VIII; E: vas deferens) or with preimmune serum (B: testis stage VII; D: stage VIII; F: vas deferens) followed by secondary antibody incubations and microscopy. Magnification: 400×.

RtMR protein immunolocalization in the testis and the vas deferens provided additional evidence of rtMR expression in male gonads in fish (Figure 2). No or very faint signals were detected after parallel incubations with preimmune serum as a negative control (Figure 2B, 2D, 2F). RtMR immunoreactivity was detected in both cytoplasmic and nuclear areas of stained cells. At stage VII and VIII, no signals are observed in sperm (Fig. 2A, 2C). By contrast, positive staining was detected around the lobules at the level of residual spermatogenesis cysts at stage VII (Figure 2A) and in sertoli and/or perilobular cells at stage VII-VIII (Figure 2A, 2C). In the vas deferens, immunohistochemistry showed positive staining along the external and internal epithelium and also in some cells of the stroma (Figure 2E).

### Effects of DOC and MIS treatments on the initiation of milt production and on sperm parameters

The effects of DOC or MIS were tested by hormone implantation into males at the end of the spermatogenetic cycle, just before the beginning of milt production (spermiation).

**Figure 3:**
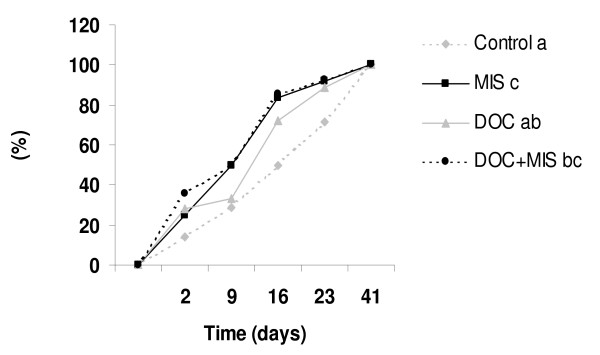
***In vivo *effect of DOC and MIS on spermiation induction**. The percentage of spermiant fish increased after implantation; with sham implants (control), MIS (5 mg/kg); DOC (10 mg/kg) or DOC (10 mg/kg) plus MIS (5 mg/kg). Means (n = 6–18). Different letters indicate significant differences between treatments, p < 0.05.

Table 2 shows the time course of plasma DOC and MIS concentration after hormone implantation, expressed relative to levels in mock-implanted controls. Over the first 16 days post-implantation DOC and MIS levels were elevated over those of controls (p < 0.01) in treated fish, and the average induction was about 5–10-fold for DOC and 2–3-fold for MIS. Figure 3 shows the percentage of males producing milt within the different groups over the time course of the experiment. Only the fish that did not produce milt at the beginning of the experiment were kept in this analysis. On the last day of the experimentation period, all experimental fish had started spermiation. As compared to the control group, DOC implantation had no significant effects on the kinetics of the initiation of milt production. By contrast, the percentage of males producing milt was significantly higher in the MIS and MIS+DOC groups than in the control group (p < 0.05).

**Table 2:** Changes in DOC and MIS blood plasma concentration after steroid implantation.

**Time (days)**	**2 (n = 4)**	**9 (n = 4)**	**16 (n = 4)**	**23 (n = 4)**	**41 (n = 4)**
**Mean DOC plasma induction**	530%*	500%*	1100%*	770%*	850%*
**Mean MIS plasma elevation**	313% *	230%	200% *	91%	81%

Values correspond to the mean in treated groups expressed as % of the mean in control groups (n = 4 per group). Asterisks indicate significant fold-induction

**Figure 4:**
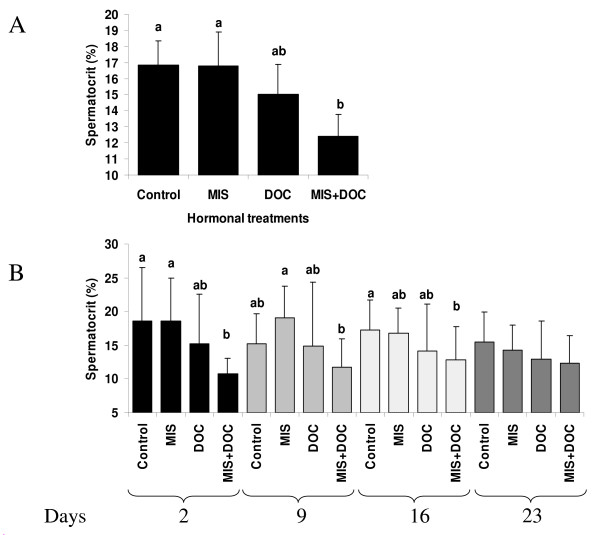
***In vivo *effect of DOC and MIS on spermatocrit**. (A) Effects on spermatocrit values of male treatments; with sham implants (Control); MIS, (5 mg/kg); DOC (10 mg/kg) or DOC (10 mg/kg) plus MIS (5 mg/kg). Each histogram corresponds to milt collected at different times post treatment. Means+ SD (n= 48–66). (B) Daily decomposition of this treatment effect. Means+ SD (n = 6–18). Different letters indicate significant differences between treatments, p < 0.05.

No significant changes in the average spermatocrit values were detected during the experimental period in all groups (no "sampling time" effect on spermatocrit). By contrast, a significant treatment effect on this parameter was observed (p < 10^-4^) (Figure 4A). In control fish, the average spermatocrit was 16.9%. DOC or MIS supplementation alone had no significant effect. However, DOC and MIS in combination induced a significant decrease of the spermatocrit as compared to control (26% decrease; p < 10^-4^). Figure 4B shows the effect of treatments at each sampling time: At days 2, 9, and 16 post-implantation, there was a significant global treatment effect on spermatocrit values (p < 0.05). While DOC and MIS alone had no significant effect, the combined DOC+MIS treatment decreased the spermatocrit significantly by 43%, 23 % and 26% respectively (effect statistically significant at days 2 and 16, p < 0.05). At day 9, the spermatocrit in the DOC+MIS group was significantly lower than in the MIS group (39% decrease, p < 10^-3^).

No statistically significant effect of the hormonal treatments could be detected on the following sperm parameters: milt volume; seminal fluid osmolality; sodium concentration or potassium concentration; sperm pH; % or spermatozoa motility. Their average values throughout the experimental period are presented in table 3. Average milt volume increased over the experimental period (p < 10^-4^). A low osmolality, with low potassium and sodium were observed in the earliest seminal fluid collected (p < 0.05). Surprisingly, we observed a slight sperm acidification (p < 10^-3^) with a sperm pH lower at day 16 than at day 2. We did not detect changes in sperm motility parameters during the first 3 sampling times.

**Table 3:** Milt parameters variations during the implantation period. Changes in average volume (ml), seminal fluid osmolality (mOsm/kg), seminal fluid sodium and potassium concentrations (Meq/L), pH and spermatozoa motility of sperm collected at different times after implantation.

**Time (days)**	**2**	**9**	**16**	**23**	**41**
**Volume (ml)**	2.3 ± 1.2 a	2.9 ± 2 a	3.2 ± 2.9 ab	4.3 ± 3.2 b	5.3 ± 3.6 c
**Seminal fluid** ** Osmolality (mOsm/kg)**	192 ± 33 a	220 ± 43 ab	230 ± 38 ab	216 ± 41 ab	234 ± 46 b
**Seminal fluid sodium** ** Concentration (Meq/L)**	78 ± 17 a	99 ± 19 b	99 ± 17 b	89 ± 16 ab	98 ± 26 b
**Seminal fluid potassium** ** Concentration (Meq/L)**	21 ± 9 a	24 ± 6 ab	25 ± 6 ab	27 ± 6 b	28 ± 6 b
**PH sperm**	8.19 ± 0.19 a	8.1 ± 0.16 ab	8 ± 0.2 b	-	-
**Spermatozoa motility (%)**	69 ± 34	66 ± 33	77 ± 20	-	-

Values are means of all experimental groups ± SD (n = 24–55). Different letters indicate significant differences between sampling dates, p < 0.05.

### *In vitro *effects of corticosteroids on 17,20β-P production

**Figure 5:**
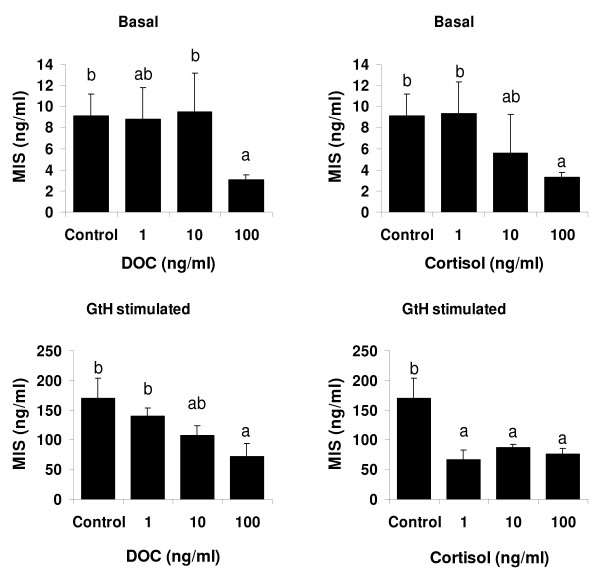
***In vitro *effects of DOC and cortisol on gonadal MIS production**. MIS concentration after a 24-hour *in vitro *incubation. Testis fragments were incubated in the presence of different steroids: cortisol (1, 10, 100 ng/ml); DOC (1, 10, 100 ng/ml) incubated with or without GtH (30 ng/ml). Ethanol vehicle represents the control. Means+ SD (n = 3–6). Different letters indicate significant differences between doses, p < 0.05.

The effect of cortisol and DOC (1, 10 or 1000 ng/ml) on the *in vitro *production of MIS was studied using testis fragments from milt-producing fish (Figure 5). DOC and cortisol added at high concentration were both effective to induce a significant drop in MIS basal production (66% and 63% respectively) (p < 0.05), while 10 ng/ml cortisol had an intermediate effect. In the presence of a salmonid gonadotrophin preparation, the *in vitro *MIS production was increased about 17-fold. In these GtH stimulated conditions, cortisol significantly inhibited MIS production at doses from 1 ng/ml (49–61% decrease) (p < 0.05) and DOC had a dose dependant effect, with a decrease of MIS production by 58% observed at the dose of 100 ng/ml (p < 0.05).

## Discussion

In teleost fish, the control of the spermiation process (hydration of semen and release by sperm duct) and the maturation of spermatozoa is only partly understood. The present study explored the potential involvement of 11-deoxycorticosterone (DOC), a mineralocorticoid, on the male reproductive tract function at the time of hydration and excretion of milt, in rainbow trout *Oncorhynchus mykiss*, a cyclic teleost fish.

We show that DOC plasma levels are particularly high around the time of spawning in mature male *O. mykiss *(Figure 1). DOC plasma levels peak around November in an autumn spawning strain and around May in a spring spawning strain, demonstrating that these changes are not "seasonal" variations. To our knowledge, this is the first report of plasma DOC up-regulation (10–50 fold) during a transitory period at the end of the male reproductive cycle in any teleost species. DOC has previously been reported in the blood plasma of one mature male *O. mykiss *and in mature winter flounder *Pseudopleuronectes americanus*, but in these studies DOC was not monitored in immature fish or outside the spawning period [27,35]. Interestingly, this steroid was found to increase 38 fold in the blood of *Tilapia aurea *females after the initiation of spawning to reach values up to 22 ng/ml [36]. By contrast, DOC was not detected in ovulated common carp *Cyprinus carpio *[37] or in pre-spawning Pacific hagfish *Eptatretus stouti *and sea lamprey *Petromyzon marinus *[38], and no difference in DOC plasma concentration was detected between gravid and sexually regressed female catfish *Heteropneustes fossilis *[39]. These inter-species differences require further investigation. Nevertheless, the progressive increase in DOC observed here at the beginning of the spawning season means that we cannot rule out a direct or indirect role of DOC during trout spermiation.

In this study, plasma 17alpha, 20beta-dihydroxyprogesterone (MIS) also rose dramatically at the onset of spermiation and in close relation to the spermiation index (Figure 1). This confirms previous data [6,19]. In the mature male population, peak values of the average DOC blood plasma levels were observed when all mature males had just started to excrete milt, and just before the peak values of MIS. The elevation of MIS and DOC coinciding with milt release is in agreement with the hypothesis that both steroids could be involved in sperm production or maturation in trout.

In mammals, the mineralocorticoid receptor (MR) main function is to regulate ionic homeostasis [20]. The *O. mykiss *MR mRNA was detected in the spermiduct and in the testis at all studied reproductive stages (Figure 1). The relative abundance of this transcript was lower in mature testis compared to immature ones, probably due to a dilution effect linked to a decrease in the somatic cell/germ cell ratio during maturation. Such dilution effect has previously been observed for proteins or transcripts expressed in the testicular somatic cells [28,40,41]. Indeed, immunohistochemistry localized the rtMR to cells at the periphery of the seminiferous tubules (Sertoli and/or peritubular cells), but germ cells did not express the receptor (Figure 2).

At the end of the cycle, we further demonstrated an increase of testis rtMR mRNA abundance, particularly in mature males at the beginning of spermiation, just after the rise in plasma DOC (Figure 1). This observation is interesting since DOC strongly activates rtMR *in vitro *[26] and therefore is a potential ligand of the rtMR. The apparent relation between DOC plasma level and rtMR mRNA abundance is the first observation suggesting a physiological link between DOC and the rtMR. But, proving that DOC is the rtMR physiological ligand, the low plasma DOC level measured in immature fish would raise the question whether DOC is able to activate the rtMR in this physiological state. In that way, the higher rtMR mRNA observed in immature fish might increase the gonadal receptivity for DOC. Further studies are needed to understand DOC action outside the reproduction period.

An expression of MR has been described in mammalian testis, and the receptor has been localized to Leydig and Sertoli cells [42]. This study is the first to report the cellular localization of MR in the reproductive tract of a fish species. Our immunolocalization of rtMR shows that mineralocorticoids could act on Sertoli cells and/or peritubular cells, which would be consistent with the involvement of these cells in water and ionic exchange with the germ cell compartment. In addition, presence of rtMR along vas deferens epithelium further argues for its implication in sperm hydration and ionic composition. Indeed, during fish spermiation, the sperm excretion is accompanied with important aqueous and ionic exchanges in the testis and the vas deferens [43,44]. Variation of rtMR expression and localization during spermiation suggest that the rtMR might be involved in the endocrine control of this process.

In this study, the elevation of DOC and MIS coinciding with milt release is in agreement with the hypothesis that both steroids are implicated in trout spermiation. We tested the influence of DOC and MIS *in vivo *supplementation on several sperm parameters. In the present study, the initiation of spermiation was shown to be significantly advanced by MIS (Figure 3). In agreement with our observation, injections of MIS also induced precocious spermiation in amago salmon and brook trout [11-15]. The rather limited effect of MIS in our study might be explained by the relatively small increase of blood plasma levels of MIS obtained with the implants (from 0.7 ng in controls to 5 ng/ml, compared to the blood concentrations of 40 – 50 ng/ml observed *in vivo *during spermiation). In our study, DOC supplementation alone was not effective to trigger initiation of milt excretion, and did not appear to act synergistically with MIS on this parameter. This observation leads us to think that DOC itself is not strongly involved in the initial induction of milt release.

In previous studies, treatments with GnRH or MIS have induced an increase in milt volume in different fish species [2,3,10,16,45]. In our hands, neither DOC nor MIS significantly increased sperm volume in *O. mykiss*. We cannot exclude that the lack of effect is related to the specific experimental conditions of this study, but MIS injections have been found ineffective to increase milt volumes also in spermiating males of other salmonids [18,19].

While Baynes and Scott [6] have found some positive correlations between blood plasma MIS levels and seminal fluid sodium/potassium concentrations in *O. mykiss*, MIS administration did not stimulate ion transport in the vas deferens in a previous study with brook trout [15]. In our experiment, no effect of DOC and MIS treatments was observed on osmolality or sodium/potassium concentrations in the seminal fluid, and the correlations reported by Baynes and Scott [6] remain to be explained. Finally, in some teleosts it has been suggested that progestin receptors exist in spermatozoa, and a direct effect of progestins on a sperm carbonic anhydrase and in the increase of pH in seminal plasma has been proposed that could be involved in sperm maturation [46,47]. However, results obtained in our study do not support a strong effect of DOC or MIS on the seminal fluid pH and spermatozoa motility.

Despite the absence of a significant effect on milt volume, DOC and MIS together significantly reduced the spermatocrit, whereas individually they did not (Figure 4). *In vivo *treatments with MIS in sea plaice *Pleuronectes platessa*, Atlantic halibut and Japanese eel *Anguilla japonica *support the role of MIS in the hydration of the milt [14,16,17]. In our *in vivo *study, MIS supplementation alone was not effective. This may have been due to the fact that only a moderate increase of this steroid in blood plasma was induced. However, our results suggest an interaction between DOC and MIS, resulting in increased milt hydration. To test whether this could possibly involve an indirect effect of DOC through a modulation of steroidogenesis, we investigated this possibility by studying MIS production in a tissue explant system. Cortisol, known to inhibit androgen production (see below), was also tested. It revealed that, *in vitro*, high concentration of DOC inhibited basal and LH stimulated MIS production (Figure 5). Interestingly, cortisol, within a physiological range of concentrations, also strongly reduced MIS production. Exposure to stress or cortisol has been shown previously to disrupt reproductive processes in fish, in particular by depressing gonadal steroid hormone levels [48-51]. To our knowledge, this is the first demonstration of cortisol and DOC effects on MIS production. It should be noted that DOC is a precursor of cortisol in fish; thus it could influence MIS production directly, or act after being metabolized into cortisol. With respect to the latter possibility, it is worth noting that 11beta-hydroxylase, which is the final enzyme of cortisol biosynthesis, shows high expression levels in testis [52]. On the one hand, our observations refer that corticosteroids and MIS signalling pathways could interact during gonad final maturation [53]. On the other hand, however, if MIS has a stimulatory role in spermiation, the down-regulation of MIS by DOC does not readily explain a positive role of DOC in this process. An alternative explanation, based on the immunolocalization of rtMR in this study and our previous finding that DOC is a strong agonist of the rtMR *in vitro *[25] is that DOC possibly acts via the rtMR directly on the seminiferous tubule and the efferent duct epithelium, affecting mechanisms related to water and ion exchange. To further elucidate the mechanism of DOC actions in the male teleost gonad, it would be interesting to investigate the potential involvement of the enzyme 11beta hydroxysteroid dehydrogenase 2, which metabolizes cortisol into cortisone, thus potentially allowing DOC to access the MR despite the greater abundance of cortisol in plasma. Finally, we speculate that DOC's effects during spermiation could also be mediated through a pathway involving a nuclear receptor similar to the one that was characterized in seatrout ovaries and showed high specificity for C21 progestagens and 11-deoxycorticosteroids [54]; however, such a receptor has not yet been characterised in the *O. mykiss *testis.

## Conclusion

High levels of plasma DOC occured in male rainbow trout *O. mykiss *around the time of spermiation. Furthermore, we have detected MR expression in the testicular tissue and the vas deferens, and have found an increase in MR mRNA abundance in mature testis during the initiation of sperm production in *O. mykiss*. In male *O. mykiss *supplemented with steroid hormones, MIS but not DOC promoted the initiation of spermiation, whereas DOC and MIS treatments synergized to decrease the spermatocrit value and therefore increase sperm fluidity. Finally, we demonstrated an *in vitro *regulatory effect of DOC and cortisol on the testicular tissue that results in changes of production of MIS, a progestin involved in the regulation of sperm production and maturation. Together, these results support the notion that DOC, an agonist of the *O. mykiss *mineralocorticoid receptor, could have roles during *O. mykiss *spermiation, which could concern the control of milt fluidity/hydration. That would be the first role proposed for this mineralocorticoid in fish. The mechanisms of action potentially involved are still speculative.

## Competing interests

The authors declare that they have no competing interests.

## Authors' contributions

SM and FLG carried out the *in vivo *studies (steroid implantation, tissue collection, milt parameters measures). AS developed the rtMR antibody. SM and XT carried out the rtMR immunohistochemistry. FI, FG and JF carried out the plasma DOC assay development and the measures. The *in vitro *work was done by SM and FLG. SM wrote the manuscript. FLG and PP managed the project and AS, FLG and PP revised the manuscript. All authors read and approved the final manuscript.
